# Defining the Digital Self: A Qualitative Study to Explore the Digital Component of Professional Identity in the Health Professions

**DOI:** 10.2196/21416

**Published:** 2020-09-29

**Authors:** Brandon Ruan, Yusuf Yilmaz, Daniel Lu, Mark Lee, Teresa M Chan

**Affiliations:** 1 McMaster Education Research, Innovation, and Theory (MERIT) Faculty of Health Sciences McMaster University Hamilton, ON Canada; 2 Department of Medical Education Faculty of Medicine Ege University Izmir Turkey; 3 Psychiatry Residency Training Program Department of Psychiatry University of British Columbia Vancouver, ON Canada; 4 McMaster Program for Faculty Development / Office of Continuing Professional Development Faculty of Health Sciences McMaster University Hamilton, ON Canada; 5 Division of Emergency Medicine / Division of Education and Innovation, Department of Medicine Faculty of Health Sciences McMaster University Hamilton, ON Canada

**Keywords:** professional identity, social media, digital identity, health care professionals, e-professionalism

## Abstract

**Background:**

Recent medical education literature pertaining to professional identity development fails to reflect the impact social media has on professional identity theory. Social media is transforming the field of medicine, as the web-based medium is now an avenue for professional development and socialization for medical students and residents. Research regarding identity development in social media has been primarily confined to electronic professionalism through best practice guidelines. However, this neglects other potential aspects pertinent to digital identity that have not yet been explored.

**Objective:**

This study aims to define the properties and development of the digital self and its interactions with the current professional identity development theory.

**Methods:**

A qualitative study was conducted using thematic analysis. A total of 17 participants who are social media education and knowledge translation experts were interviewed. The initial participants were from emergency medicine, and a snowball sampling method was used following their respective web-based semistructured interviews to enable global recruitment of other participants from interprofessional disciplines. The research team consisted of a diverse group of researchers including one current social media knowledge translation physician clinician educator, one postdoctoral researcher who is regularly engaged in social media knowledge translation, and 3 nonphysician research assistants who are not social media users. Half of the team conducted the initial coding and analysis, whereas the other 2 investigators audited the procedures followed.

**Results:**

A total of 4 themes were identified that pertain to digital identity. In the first theme, origins of initial digital identity formation were found to be derived from perceived needs in professional roles (eg, as a medical student or resident). The second theme consisted of the cultivation of digital identity, in which digital identity was developed parallel to professional identity. The third theme that emerged was the management between the professional and personal components of digital identity. Participants initially preferred keeping these components completely separate; however, attempts to do so were inadequate while the integration of both components provided benefits. The fourth theme was the management of real-life identity and digital identity. Participants preferred real-life identity to be wholly represented on the web. Instances of misalignment resulted in identity conflict, compromising one of the identities.

**Conclusions:**

Social media introduces new features to professional identity in the digital world. The formation of digital identity, its development, and reconciliation with other identities were features captured in our analysis. The virtual component of professional identity must not be neglected but instead further explored, as educational institutions continue to give more importance to navigating professional identity development.

## Introduction

Professional identity has become an increasingly prominent phenomenon that is thought to be crucial to consider when educating health profession trainees [1-5]. Professional identity development is a recurring and adaptive process; personal identity that is constructed by one’s internal values and morals molds and projects into the formation of the professional self, which encompasses role expectations and professional ethics [6]. For medical education, facilitating the strengthening of physician identity and internalizing physician values bring confidence into clinical practice and creates humanistic, compassionate, and ethical physicians [1,4]. The medical education literature has proposed models for navigating professional identity development and has documented its resiliencies [7,8]; however, recent work in this area does not explore the impact of new forms of expression, such as social media, on professional identity formation or development.

Opportunities for professional development through collaboration and networking have become more accessible, as social media platforms such as Twitter facilitate more continuous communication [9,10]. Furthermore, medical education literature suggests that the digital space also acts as an avenue for informal learning and socialization for medical trainees [11-13]. To navigate these spaces effectively, best practice guidelines have been published [14-18]. However, these guidelines often focus on confining trainees to a professional frame, often with an emphasis on preventing lapses in professionalism [19-21].

With social media altering the methods of learning, communication, and collaboration, it is imperative to investigate how professional identity fully manifests in the digital space. To continue guiding the next generation of physicians through professional identity development in an era of social media, institutions must aim to train them in the growing digital space in conjunction with the physical space.

Although there is an importance in practicing appropriate professional conduct on social media, continuously expressing the *professional self* may conflict with personal identity development—forcing an intricate interplay between personal identity and the professional self in an open forum that has previously been impossible [22]. It is within this area of tension that the components of professional identity may manifest uniquely in the digital space and that the establishment of one’s professional self within a digital world may currently be unexplored.

The purpose of this study is to explore the digital component of professional identity: digital identity. We aim to extend the literature by defining the characteristics, properties, and development of digital identity and its interactions with the current professional identity development theory.

## Methods

### Research Paradigm

Through interviewing research experts and collecting their responses, we conducted a qualitative study to identify the underlying themes and phenomena guiding identity development using generic thematic analysis [23]. We applied an interpretivist lens to examine the recollections of our participants as they pertained to their personal and professional identities.

### Research Team

The research team comprised 5 individuals: 1 physician (TMC) experienced in knowledge of the background literature on social media, teaching, and learning and in qualitative analysis, a postdoctoral researcher (YY) who is regularly engaged in social media knowledge translation and trained in qualitative analysis, and 3 nonphysician research assistants (BR, DL, and ML) trained in qualitative analysis but are not social media users. Half of the research team participated in the interpretation and analysis of the transcripts (TMC, BR, and DL), whereas the other half of the research team conducted an audit trail of the analysis and procedure (YY and ML).This dynamic of the research team was constructed to require the lead supervisor (TMC) to justify her interpretations of the themes to the other members (DL and BR) who hold no stake in the social media, teaching, and learning spheres.

### Context

The targeted context pertinent to this study was the web-based community of social media–based educators and knowledge translation experts who are engaged in health profession education disciplines. The study initially started with social media educationalists from the emergency medicine field; however, the snowball sampling technique enabled nominations from interprofessional fields outside of emergency medicine [24]. The initial selection of web-based knowledge translation experts from the emergency medicine field was justified owing to their likelihood of having substantial commentary and experience on their digital identity origin and development based on the influential following they have built [25].

### Sampling

Recruitment was initiated using a randomized selection of 10 physicians from a previously published list of the top 100 most influential emergency medicine physicians on Twitter [25]. Initial participants were contacted via institutional email or social media to participate in a semistructured interview that pertains to the individual’s social media activities and origins of social media scholarship. A snowball sampling technique was followed upon completing the initial participants’ interview [24]. As the use of social media by knowledge translation experts and educators is rapidly evolving, the list by Riddell et al [25] may not fully capture the current social media sphere, therefore making the population ill-defined and an approach of snowball sampling to be appropriate [24]. The snowball sampling technique serves to better define our population of interest by requiring participants to identify and nominate other potential influencers within their social networks, who are also likely to have extensive experiences regarding digital identity origin and development. In the context of our study, the snowball sampling technique also enabled nominations from interprofessional fields, allowing us to capture social media knowledge translation experts in the health profession education disciplines beyond emergency medicine. Nominations were requested upon the completion of each participant’s interview until thematic sufficiency was reached.

### Ethics Approval

Ethics approval was granted by our institutional review board, the Hamilton Integrated Research Ethics Board (HIREB-5609).

### Data Collection: Methods and Analysis

Interviews were conducted by the research assistants (BR and AM). The research assistants were trained in semistructured interviewing through simulations with the project lead. Semistructured interview prompts were constructed into the interview guide (Multimedia Appendix 1), and adaptations owing to missing gaps within the delivery of these prompts were made after the initial transcripts were reviewed. Participants did not review transcripts following their synthesis. All interviews were conducted and recorded using the Zoom (Zoom Video Communications, Inc) calling interface version 4.5.6, with audio being recorded [26].

### Data Processing

Interviews were recorded, and the audio files were sent to a third-party professional medical transcriptionist. Transcripts were verified or corrected, if required by the interviewer and the analysis team, to confirm the accuracy of the transcripts.

### Data Analysis

The research team reviewed the transcripts using a generic thematic analysis, deriving themes related to the identity formation of the participants. A total of 17 transcripts were obtained and analyzed for themes, 2 to 4 batches at a time over several months. Each coding session outputted a codebook that was saved, archived, and updated for the next session. The codebook was updated and restructured every session until thematic sufficiency was reached in concepts pertinent to digital identity.

### Enhancing Rigor and Trustworthiness

Following the completion of transcript analysis, an audit was conducted by YY and ML to assess our analysis trail. Both auditors were given the full codebook and archives in addition to full primary transcripts. Owing to the ongoing COVID-19 pandemic [27,28], and the demographics of our participants being frontline health care workers, the initial plan to conduct a final member check of our analysis was revised. This study on digital identity is an umbrella study that consists of a larger project with 2 phases of interviews. For this particular study, only the first phase of the interview data was used for analysis. The division of this study was defined as *a priori*. Data analyses were separated to ensure that each unique component of the umbrella study reached data sufficiency. We adhered to the *Standards for Reporting Qualitative Research* checklist for reporting [29].

## Results

### Demographics

A total of 17 international social media experts were recruited and interviewed (male, 10/17, 59%; female, 7/17, 41%). Data surrounding their personal and professional identities were collected, including their Twitter followership. The mean interview duration was 30.6 (SD 7.98) min. The range of interview length was 18.6 min to 52.1 min. A total of 189 pages of transcripts were analyzed and reviewed. Most interviewees were partnered with dependents (9/17, 53%) or single with no dependents (5/17, 29%). Details on participant demographics are shown in Table 1.

Countries of academic or clinical practice included 47% (8/17) from the United States, 29% (5/17) from Canada, 12% (2/17) from Australia, and 12% (2/17) from other countries. The participants also varied across academic ranks: 35% (6/17) were assistant professors, 24% (4/17) were associate professors, 18% (3/17) were adjunct scientists, and 6% (1/17) were assistant professors. All participants identified their academic roles as educators (17/17, 100%). In addition, most participants also identified their academic roles as either a clinician (15/17, 88%) or a research investigator (13/17, 77%).

A multitude of social media platforms were identified by interviewees for use in both personal and professional contexts. The personal use of social media consisted of a majority that use Twitter (14/17, 82%), Facebook (14/17, 82%), and WhatsApp (11/17, 65%). In conjunction with their personal use, Twitter was also identified by all the participants for professional use (17/17, 100%), and most participants used Google Scholar (10/17, 59%). All social media platforms identified by interviewees for personal or professional use can be found in Table 2.

Major themes pertaining to digital identity are summarized in Table 3 and further elucidated below.

**Table 1:** Participants’ demographics.

Demographic characteristics		Values	
**Gender, n (%)**			
	Female		7 (41)
	Male		10 (59)
**Characteristics of family and caregiving, n (%)**			
	Single, with no children or dependents		5 (29)
	Single, with children or dependents		1 (6)
	Partnered, with no children or dependents		2 (12)
	Partnered, with children or dependents		9 (53)
**Social media presence, median (IQR)**			
	Number of Twitter followers		6800 (3391-10,500)

**Table 2:** Frequencies of social media platforms for personal and professional use.

Social media platform used	Frequency	
	Personal use, n (%)	Professional use, n (%)
Twitter	14 (82)	17 (100)
Facebook	14 (82)	8 (47)
Google Scholar	10 (59)	10 (59)
WhatsApp	11 (65)	6 (35)
Slack	7 (41)	8 (47)
LinkedIn	7 (41)	7 (41)
ResearchGate	5 (29)	8 (47)
ORCID	5 (29)	8 (47)
Instagram	9 (53)	3 (18)
Reddit	3 (18)	3 (18)
Snapchat	2 (12)	1 (6)
Academia.edu	1 (6)	2 (12)

**Table 3:** Major themes in the construct of digital identity.

Themes	Summary	Representative quote
Initial formation of the digital identity	Most participants identified an initial hook into social media when starting their digital identity	“So, when I started off on social media, I was only on Twitter. I actually got into social media as almost a bet.”
Cultivating digital identity	Digital identity evolves rapidly and can develop in parallel to professional identity	“Yeah, I guess my identity has changed on social media as my [professional] identity has changed throughout my training.”
Real-life identity versus digital identity management	Participants noted the importance of representing themselves holistically on the web	“I am who I am. And I feel comfortable being who I am and representing myself, as such.”
The professional and personal dimensions of social media	Some participants attempt to separate their professional and personal identity on the web; however, others demonstrate convergence is inevitable	“So, I used to keep things more separate and then and as my presence in social media has evolved, they have tended to grow closer together both the personal and the professional and merge into one because well that is who I am…”

### Initial Formation of Digital Identity

Participants were asked to identify their origin of digital identity and how they began engaging in social media. Identified incentives guiding their initial use of social media were categorized into the following: (1) meeting an educational need and (2) facilitated via role responsibilities. These incentives acted as a hook for our participants into social media, as they continued their web-based activity once initialized into the digital space.

#### Meeting an Educational Need

Participants highlighted the educational value found in social media resources in their workplace environment:

I think [social media] now also supports structured education in the emergency department as well as just-in-time practice...

Participant F1

Other participants who harnessed social media as an academic resource utilized it during their professional identity as a medical student. In one participant’s experience, social media initially aided in further bolstering engagement with their academic lecture and conference material:

Honestly, I started as a way to pay attention during lectures and conferences while staying engaged. It was a tool to essentially take notes. And do something that would kind of keep my ADD at bay while actually still engaging in the talks.

Participant M1

As Participant M1 continued to immerse into social media, their use of social media transformed outside their initial intentions from an academic tool into an avenue to share and disseminate content:

...and then that became a good way, also I found that it was also a good way to kind of share things that I was learning.

Participant M1

Another participant expressed social media as a platform for engagement despite their early hierarchical status as a medical student. Moreover, social media satiated their desire to engage with the community of practice in the early professional development stage as a medical student:

I was just a medical student...I think initially I was just excited and overwhelmed by the number of resources that were available and the number of people who were willing to engage with me as a medical student. And so, I was probably mostly excited about content at that phase and using [social media] to consume content.

Participant F2

#### Role Responsibilities

Other participants identified their origin of digital identity being formed through various pressures related to role responsibilities directed by supervisors. In these instances, it was their supervisors or superiors who engaged with our participants that encouraged them to start their digital identity:

My program director had asked that I start writing weekly pearls, the [Residency Program] pearls are still generated but that got me in the habit of writing something in an electronic format weekly.

Participant M2

Another participant experienced unconventional pressures from their supervisors through competition and boisterous wagers that ignited their digital identity’s birth. In such cases, the result of long-term social media activity still prevails:

I made a bet with the VP who was in charge of operations and public relations.I said that in three months that I could have a bigger following than the hospital system and as a physician. And I think it took me five months. But I definitely did get more and still have more followers than the hospital system.

Participant F3

### Cultivating Digital Identity

#### Socialization Into the Web-Based Community of Practice

As our participants engage further into the digital realm, the norms of the digital space are learned. By learning these rules, the digital identity of the participants begin to solidify. Participants are also able to increase their precision in navigating the digital space, allowing them to recognize which content garners them a stronger following. Resulting from this are networking opportunities and further engagement in the community of practice, as mentioned:

I made an account and very soon after six months I went to the #1 [spot]...I created an infographic because I just wanted to see if I could make one. And I put it out there and it got...300, 400, then 500 likes [and] retweets...So, it’s like people started seeing my name. And then I got asked to do some training and talks: like hey you are good at this...And it really just snowballed.

Participant F4

#### Growth Via Parallel Development

The development of digital identity was noted by Participant F2, who explained that, in their experience, digital identity developed parallel to their professional identity development in real life. Namely, one participant’s (F2) identity from being a medical student to being a resident and her progressive development in expertise was similarly reflected in her digital identity, as she explains:

...So, I think my position in the community has changed as I have developed more expertise and figured out really who I am as an academic and as a member of this community.

Participant F2

#### The Characteristics of Digital Identity: Fluid and Dynamic

Many participants expressed the different roles digital identity manifested, including “doing more critical appraisals and reviews” to “educating followers on areas of expertise” or “sharing [your] own work.” Interestingly, despite the many roles that exist in the academic digital space, they are not singular nor mutually exclusive, as participants noted their “role as having multiple components.” The evolution of digital identity was identified by participants to be fluid, transitive, and dynamic, where roles could be added over time or compounded:

Yeah, so I think there are a few different roles, so one is as a producer of content for social media and that is in our podcast... I also [identify] myself as an occasional contributor to other social media sites. And then I see myself as a consumer and sometimes curator.

Participant F1

### The Professional and Personal Dimensions of Digital Identity

#### Desire to Be Professional

When we asked our participants about their contexts of social media use, many responded “primarily using it professionally” to the public. Other participants preferred a defined “dichotomous approach,” separating professional use from personal use on social media utilizing strategies such as “using different platforms” for different identities. The observed management of identity separation on social media demonstrates an implicit expectation to confine to a purely professional image on the web.

#### Challenges to Disentangle Personal and Professional

Although some participants expressed the importance of maintaining a professional public frame on social media at all times, other interviewed experts expressed challenges in doing so. When browsing through social media content on platforms such as Twitter, one comes into contact with various types of content to which “you can’t help but come across things that are of interest and not strictly professional.” Blurring the lines between the personal and professional components of digital identity also extended to colleague relationships as elucidated by one of our male participants (M3):

I definitely started with very strict bounds where my only content that I really used personally was Facebook and everything else was professional. But that is essentially impossible to keep up because as you spend more time professionally [on social media], you become really good friends with a number of your colleagues. And therefore, they become part of your personal circle.

Participant M3

#### Convergence Is Inevitable

The active divide in splitting professional and personal components of digital identity is ineffective. Even common strategies such as explicitly stating the distinction of accountability in the profile biography between the professional and personal identity are viewed by our participants to be insufficient. This strategy of explicitly stating this distinction is intended to safeguard organizations from personal stances and behaviors that may not align with the organization’s values and professionalisms; however, one of our male participants criticized as follows:

People like to put disclaimers in their Twitter bio saying that they don't represent their employers. I think that [it] is kind of nonsense. It is not going to help you legally if you get into trouble with your employer. It is not a get out of jail free card. So, I do mention where I work. And so far, that hasn't caused any problem with my employer.

Participant M4

#### Benefits of Convergence

Our interviewed participants have given diverse responses when managing personal and professional identities on social media; however, participants like M4 have experienced no consequences integrating both. Furthermore, researchers (such as F5) bolster their professional practice by harnessing their personal identity on the web:

So, with Twitter, I don't really have the separation because it is more about the sort of medical, networking side of things and a little bit less about my very personal experience of the world. Although there is a bit of that [personal experience] as well. And in my opinion, my experience is enhanced by being by presenting myself as a human and having a kind of complex life outside of academia or pure clinical roles.

Participant F5

### Real Life and Social Media Alignment

#### Observation of the Alignment

When asking participants about their behavior and identity in the digital space with respect to real life, most participants expressed an alignment between these identities. One participant engaged with other scientists on the web as if they were their colleagues in real life:

I just have normal conversations as I would if I was having a conversation at a conference or some other professional context with people.

Participant M5

As expected, such an alignment further extended to academic interests and roles as well, demonstrating social media as an extension to developing the identity of these researchers. In one participant’s case (M6), their interest in medications and advocacy for safe and established treatments directed to the scientific community reflected their real-life academic identity:

One of my main interests is medications, and so I would like to encourage what I perceive to be safer drug therapy. I also like to give followers insight into clinical cases or also sometimes even personal stuff.

Participant M6

#### Importance of Holistic Representation

Participants identified an inherent importance of centralizing their digital identity to their true identity in real life. One participant highlights a state of comfort when representing their full digital identity by balancing their professional and personal identity on the web as they refuse “artificially keeping [professional and personal identity] separate.” Another participant supported this view in avoiding artificial separation of personal and professional identities when engaging with other educators on the web*:*

I don't generally have interactions that I wouldn't have on professional contacts on my Twitter channel, which as I noted I use for personal and professional purposes.

Participant M5

#### Consequence of Misalignment

When immersing further into the community of practice, opportunities for misalignment between the physical and digital identity may occur in which the physical identity lags:

It has taken a while for my [online identity] to also become reflective in my public identity”

Participant F6

In another participant’s case, the rift was formed because of a noted, enhanced capability of networking on social media:

I feel like I occasionally get some type of imposter syndrome by this group of people online and that is the network of people that I have met online and have worked with on a number of projects. I think they see me as really valuable and this person that they want to work with and really value what I bring to the table. I don't always feel that same way at my home institution that I work at.

Participant F2

In this instance, where digital identity outpaces the physical professional identity, the misalignment of these identities caused this participant to compromise or restructure one of her identities to fit the latter:

And so, then I wonder: is this a bit of a disconnect between who I am in real life and who I am online? Or is it actually that there are people in other organizations that actually value what I bring to the table a little bit more than where I am at.

Participant F2

## Discussion

Through the collected experiences from our interviewed social media experts, our team has investigated the surrounding interactions and explored the construct of a digital identity. For our experts, the initial formation of digital identity materialized through needs present in the early stages of professional identity in medical students. Proceeding initial growth, the development of digital identity molded in conjunction with real-life professional identity development and expertise. The importance of representing oneself holistically on the web between the physical and digital identities and between the personal and professional identities was highlighted.

Our main finding is that the fluid and dynamic nature of digital identity are similar to those of professional identity’s fluidity [30-32]. Participants often transformed, even interchangeably, through different roles from education bloggers, critical appraisers, or even extending to personal characters. Interestingly, the transitive property of easily interchanging roles in our findings of digital identity contrasts the disruption faced when frequently transitioning between different professional identities [33]. Our analysis also captured the behavior of models by Lave and Wenger [34] on situated learning and community of practice. Engagement at the initial identity formation stage starts peripheral as our participants begin developing their content [34]. Through the feedback process of socialization of continually deeper engagement, the identity becomes more refined until becoming a member of the community of practice [34]. In the case of our findings on digital identity, web-based metrics such as followers or increased web traffic were successful markers of socialization into the digital community and guided our participants to refine their digital identity by adhering to engagement strategies [35] and content that increased followings.

Although similarities exist between the characteristics of digital identity and professional identity development, differences are highlighted through the pace of development via the digital space. Social media has overcome physical barriers and allows health professionals to have a global reach to engage with national committees and remote stakeholders [12,21,36-39]. This allowed our interviewed participants who faced physical barriers such as practicing rurally to otherwise come across opportunities that normally would not exist. Occasionally, identity mismatch would occur as professional identity lags behind digital identity development.

To resolve the tension between identity mismatch, it was found to be important to capture a holistic identity and represent it digitally. This finding contrasted with identity management techniques observed by Cho and Jimerson [39], where professional identity was publicly expressed whereas personal identity was compartmentalized in separate social media accounts. Their study additionally found that professional identity was further fragmented between different organizational social media accounts as an engagement strategy to tailor to different audiences [40]. These differences between our findings may highlight deviations in identity management strategies between organization and individually owned social media accounts. Although the participants in the study by Cho and Jimerson [39] responded to organizational and administrative needs, our participants utilized social media to collaborate with the community of practice to further advance their scholarly work. These interactions were preferred to be organic, utilizing a natural blend between personal and professional identities. Such a preference for convergence aligns with findings by DeCamp et al [22], who argue that physicians are not intended to avoid personal interactions, as it is sometimes unavoidable. Decamp et al [22] further affirm that appropriate personal engagement on the web can bolster web-based professional interactions, to which our participants agree.

Our findings showed that digital identity formation initially begins because of a professional or academic need that can take the form of a medical student seeking to understand a specialty [7,21], or a new leader looking to connect more with stakeholders. Many of our interviewed participants explained that their digital identity began while they were concurrently developing their professional identity as trainees. This finding is in line with previous literature; a qualitative study interviewing medical students demonstrated their desire to be given a voice and engage with the online community of practice [12]. Other studies extend this narrative, showing that engagement in social media enables networking, mentorship, and content learning [36,37,39,41-44]. As Mather et al [45] highlight the benefits social media provide call the need for educational institutions to engage and facilitate the development of professional identity for learners within the digital space.

The impact of social media as a disseminator of health-related information and misinformation is one that has been undoubtedly demonstrated by its utilization during the COVID-19 pandemic [46-48]. Further exploration of digital identity development will allow educational institutions and organizations to cultivate physicians that are increasingly familiar with the digital space. By equipping these physicians with the tools to build web-based stakeholdership and develop their digital role as digital responders and disseminators of information on the web, physicians will be more ready to combat future threats of web-based health misinformation [46,47].

The current digital space serves as an extension to physical space, and its ability to capture and express our personal and professional identities is, thus, also extended in the same manner [49]. Our findings provide insight into the model of digital identity development and its interactions with existing literature regarding professional identity development. We present digital identity as an additional dimension to professional identity development within health care professionals. With social media’s dominant role as an accelerated medium for dissemination and identity development, further investigation of the interactions between digital identity formation upon professional identity development in individuals of various key stages including medical students, residents, and junior academic cohorts are warranted. These findings will be important to guide medical trainees and students through their professional identity formation in both the real and digital worlds.

### Limitations

This study had a number of limitations. The research lead investigator had expertise in web-based pedagogy and knowledge translation. Preventative measures were taken to reduce any skewed interpretations of participant answers and words. For example, the research assistant and transcriptionist interviewed all participants and transcribed all transcripts, respectively. In addition, all transcripts were deidentified before the qualitative analysis, preventing the lead investigator from deriving the identity of the participants. During the analysis phase, a variety of strategies were used with the knowledge that the lead investigator of the research team was an expert in social media knowledge translation with respect to the other members.

We focused on those who had established reputations and track records for their academic work and, therefore, likely skewed our interviews toward those who are further along in their web-based professional identity formation. Of note, we only had 1 senior trainee participant, and therefore, there may be some limitations in extrapolating our key findings to more junior learners. However, many of our participants had good recollections about their experiences with digital identity formation and origin during their training, which may allow for transferable findings to be relevant to educators seeking to counsel junior learners.

### Conclusions

Social media introduces new features to professional identity in the digital space. The formation of digital identity, its development, and interactions that require identity management were features captured in our study. Moreover, the fluid and dynamic characteristics of digital identity in conjunction with its accelerated capacity of growth yield differences from professional identity development that can potentially be harnessed. Navigating the identity development of young or upcoming health care professionals is a priority for institutions now and in the post–COVID-19 world. Today, digital identity can no longer be neglected. Digital citizenship can no longer be ignored as a key facet of one’s professional responsibility. If we are to effectively train the next generation of health care professionals in an era of ongoing technological development, digital identity development must be explored and supported.

## Appendix

Multimedia Appendix 1 Interview guide.

## Abbreviations

PSI: Physician Services Incorporated
