# Supplementary material for: Defining the Digital Self: A Qualitative Study to Explore the Digital Component of Professional Identity in the Health Professions
Source: J Med Internet Res. 2020 Sep 29;22(9):e21416. doi: 10.2196/21416 (PMC7556376; doi:10.2196/21416)
Supplement: Multimedia Appendix 1 [file jmir_v22i9e21416_app1.pdf]

Questions in the Interview Guide

PART 1: Initial Warm Up Questions

1. **Can you describe your current academic status (e.g. rank) and position within your organization? How does social media relate to this role?**
   - *What does your role actually entail from day to day?*
   - *Can you list any places where you use social media to enhance your role?*
2. **What do you see your role on social media as?**
   a. *What defines a "SM" platform varies:*
      i. *NA it tends to be Twitter/FB*
      ii. *Africa sees heavy usage of What's App*
      iii. *Personal blogs*
      iv. *Podcasts*
   b. *Why this platform?*
   c. *If multiple platforms, why? How do you manage everything?*
   d. *Do you engage in different platforms for "personal" vs. "professional" use? What drives that choice?*
3. **What social media platforms do you regularly engage in?**
   a. *What typical activities do you engage in when using social media?*
   Is there a theme/common thread to your typical activities?
   b. *Ex. Do you post a lot of work from your own lab?*
   c. *Sharing/critiquing of guidelines within a social group?*
   d. *Manage the social media presence for a group (ex. hospital/university department)*
4. *Do you consume content more than you produce content?*
5. Given the following descriptions, which of these roles would you say is the best descriptor of you?
   a. **Translational Teacher:** Strong, often trained, educators; they work with researchers to help with knowledge translation and getting the word out about new studies/findings. *(the PR)*
   b. **Critical Clinician**: Skilled at critical appraisal, these individuals critique and analyze new studies/findings in an open forum. *(The restaurant critics)*
   c. **Interactive Investigator**: Produce new studies/findings, while engaging with end-users to explain and receive feedback for improving their research. *(The traditional scientist)*
   d. **Skeptics:** Are openly critical of, and/or dislike FOAMed and the surge in social media as a method of rapid knowledge translation for a variety of reasons.
   a. Do you feel you fit into more than one?

PART 2: General Question for all

1. What got you started using social media?
   a. *Have you always had a strong online presence?*
   b. *Personal enjoyment/fulfilment?*
   c. *Some people see it as a good career move/way to stay current in their field*
   d. *Was it an institutional requirement/expectation?*
   e. *Have you changed the platforms you use?*

2. How much time do you spend in a given week producing content?
3. If so, how do you keep your professional and personal identities separate?
4. How do you feel social media/FOAMed has changed or impacted the field of medicine? Your field specifically?
    a. Do you think the impact has been positive or negative? Why?

PART 3: Particular Identity Questions

From here, begin asking questions specific to the role that we have identified them as; if they have self-identified as more than one, or as a different role than pre-assigned, ask the questions for those as well.

Critical Clinicians

1. How did you develop your credibility/visibility as a critic on social media when starting out?
    a. *What challenges did you face in trying to increase your online "impact factor"?*
2. Why do you engage in open-forum critical appraisal?
3. What advantages does this have over the more traditional peer-review process that make it attractive for you? Any disadvantages?
    a. *Enjoyment of real time feedback?*
    b. *Disadvantages – ex. communicating ideas properly on platforms with limited character count;*
4. Do you have anything else you would like to explain about your viewpoints?

Translational Teachers

1. What process do you use when choosing what new work you choose to disseminate?
    a. *Do you work with the same researcher group consistently?*
    b. *How do you find the work that you post/share*
2. How do you approach communicating with the much broader audience that you can reach with social media?
    a. *Everyone from senior physicians, to medical students, to the general public can access information on most social media platforms*
    b. *How do you navigate communicating effectively with this broad audience?*
    c. *Do you focus your efforts on specific groups?*
3. Do you have anything else you would like to explain about your viewpoints?

Interactive Investigators

1. Does advocating largely for your own research via social media pose any challenges?
    a. Is it difficult to balance the role of "investigator" with the role of "PR"?
    b. How do you keep these identities separate? Do you?
2. Has your involvement with social media, and real-time feedback from end users, changed your approach to research? How?
    a. *Ex. your process for developing questions, how you incorporate feedback into future work, etc?*
3. Do you have anything else you would like to explain about your viewpoints?

PART 4: Conclusion
Question:
Lastly, is there anything else you want to share with me?
